# Optimization of Surface Functionalizations for Ring Resonator-Based Biosensors

**DOI:** 10.3390/s24103107

**Published:** 2024-05-14

**Authors:** Niccolò Ardoino, Lorenzo Lunelli, Georg Pucker, Lia Vanzetti, Rachele Favaretto, Laura Pasquardini, Cecilia Pederzolli, Carlo Guardiani, Cristina Potrich

**Affiliations:** 1 FTH S.r.l., Via Sommarive 18, I-38123 Trento, Italy; niccolo.ardoino@femtorays.com (N.A.); rachele.favaretto@femtorays.com (R.F.); carlo.guardiani@femtorays.com (C.G.); 2 Center for Sensors & Devices, Fondazione Bruno Kessler, Via Sommarive 18, I-38123 Trento, Italy; lunelli@fbk.eu (L.L.); pucker@fbk.eu (G.P.); vanzetti@fbk.eu (L.V.); pederzo@fbk.eu (C.P.); 3 Istituto di Biofisica, Consiglio Nazionale delle Ricerche, Via alla Cascata 56/C, I-38123 Trento, Italy; 4 Department of Physics, University of Trento, Via Sommarive 14, Povo, I-38123 Trento, Italy; 5 Indivenire S.r.l., Via Sommarive 18, I-38123 Trento, Italy; l.pasquardini@indiveni.re; 6 Department of Engineering, University of Campania “Luigi Vanvitelli”, Via Roma 29, I-81031 Aversa, Italy

**Keywords:** surface silanization, aptamer functionalization, photonic biosensor, biomarker detection, on-chip liquid biopsy

## Abstract

Liquid biopsy is expected to become widespread in the coming years thanks to point of care devices, which can include label-free biosensors. The surface functionalization of biosensors is a crucial aspect that influences their overall performance, resulting in the accurate, sensitive, and specific detection of target molecules. Here, the surface of a microring resonator (MRR)-based biosensor was functionalized for the detection of protein biomarkers. Among the several existing functionalization methods, a strategy based on aptamers and mercaptosilanes was selected as the most highly performing approach. All steps of the functionalization protocol were carefully characterized and optimized to obtain a suitable protocol to be transferred to the final biosensor. The functionalization protocol comprised a preliminary plasma treatment aimed at cleaning and activating the surface for the subsequent silanization step. Different plasma treatments as well as different silanes were tested in order to covalently bind aptamers specific to different biomarker targets, i.e., C-reactive protein, SARS-CoV-2 spike protein, and thrombin. Argon plasma and 1% *v*/*v* mercaptosilane were found as the most suitable for obtaining a homogeneous layer apt to aptamer conjugation. The aptamer concentration and time for immobilization were optimized, resulting in 1 µM and 3 h, respectively. A final passivation step based on mercaptohexanol was also implemented. The functionalization protocol was then evaluated for the detection of thrombin with a photonic biosensor based on microring resonators. The preliminary results identified the successful recognition of the correct target as well as some limitations of the developed protocol in real measurement conditions.

## 1. Introduction

Liquid biopsy has been proven in recent years as an effective tool for non-invasive diagnosis and followup for several pathologies in the wide context of personalized medicine [1,2,3]. Sources of samples for liquid biopsy are almost all body fluids, such as plasma, urine, and saliva, which contain several components considered to be biomarkers of the physio-pathological state of an individual [4,5]. The accessibility of these samples and the recent development of suitable technologies has pushed liquid biopsy out of traditional laboratories toward point-of-care (POC) tests. POC tests combine several advantages, including ease of use, portability, low costs, and rapid results, which allows for their spread in low-income countries and in remote areas, where access to centralized hospital facilities is limited. Successful POC tests include, for example, lateral flow tests (LFTs), urine dipsticks, and glucometers, but they often lack sensitivity, in particular when low-abundance biomarkers are tested. For this reason, these assays are often combined with signal amplification technologies, such as the use of labeled molecules and suitable readers implemented on LFTs, which enhance the signal by several times (100-fold or more) [6,7]. To improve POC sensitivity, next-generation POC takes advantage of microfluidics [8,9,10], and simple detection methods such as smartphones [11] are being developed. A microfluidic-based POC testing for the detection of SARS-CoV-2 nucleocapsid proteins was reported to work in a linear detection range of 10 to 1000 pg/mL, with a detection limit of 3.1 pg/mL [12]. Another microfluidic platform was proven to detect C-reactive protein in the 0.1–10 μg/mL concentration range [13], while a POC test using an intradermal finger prick-based electronic nanosensor array was reported for direct protein quantification down to the sub-pM range [14]. In this context, POC tests based on label free biosensors are also explored, with some devices successfully detecting protein biomarkers down to the picomolar range [15,16]. Among label-free biosensors, whispering gallery mode (WGM) resonators are known for their high sensitivity, which makes them very attractive as biosensors. The optical confinement of light guaranteed by WGM resonators indeed results in high levels of accuracy, sensitivity, speed, and signal-to-noise ratio [17,18]. WGM resonators can have different geometries, including rings, disks, spheres, cylinders, fibers, rods, or toroids [15]. WGM resonators with a ring geometry are called microring resonators (MRRs). MRRs offer several advantages, such as a simplified fabrication process based on consolidated silicon technologies, which allow for low-cost and large-scale manufacturing. For these reasons, MRRs were used in this work as proof-of-concept biomarker photonic sensors.

Label-free biosensors have great potential in terms of sensitivity, but they are also greatly influenced by the heterogeneity of their sample components, demonstrating how surface functionalization plays a crucial role in selectively capturing the correct biomarkers [16,17,19]. The selective biomarker capture and the inhibition of non-specific adsorptions of other molecules present in the complex sample matrix are therefore the main objectives of surface functionalization. A large number of surface functionalization strategies do exist; some examples of such strategies are the physical adsorption by direct deposition of molecules, the covalent binding of molecular chemical groups to the sensor surface, and the non-covalent interactions with a deposited active layer [17]. Among the covalent binding of chemical groups, silanes are also widely used in the context of MRR [20]. This modification is based on the formation of covalent Si–O–Si bonds between the oxidized silicon structure and the coating substance, i.e., an organosilane with a chemical residue suitable for the binding of a specific recognition element. The most used silanes are amino- and epoxy-silanes [20], whereas here, a mercaptosilane was tested and compared to a more classical epoxysilane, showing much better performance.

Regarding recognition elements, several molecules have been proposed, such as peptides [21], aptamers [22,23], or molecular-imprinted polymers (MIPs) [24]. The most exploited class, however, is that of antibodies [22,25] or antibody fragments [19], thanks to their sensitivity and selectivity properties; however, aptamers are also emerging as convenient recognition elements. Aptamers are single-stranded nucleotide chains that specifically bind target molecules with high affinity and selectivity via secondary-structure formation. They have several advantages with respect to antibodies, including the fact that they work under more severe conditions, i.e., in environments different from physiological systems, as they are less sensitive to variations in pH, hydration, and temperature, which allows them to survive under harsh regeneration steps. Moreover, aptamers show reduced batch-to-batch variation compared to antibodies and can be modified by adding selective chemical groups and spacers at predetermined positions, allowing for substrate-oriented immobilization and limiting steric hindrance for an optimized binding [22,23]. The covalent binding of aptamers to the silanized surface was selected in this work as the most promising functionalization strategy for MRR biosensors.

The main aim of this paper is the development and characterization of a suitable surface functionalization for MRR structures aimed at biosensing. The different steps of the functionalization protocol were preliminary set up on plane surfaces resembling the surface chemistry of the of the MRR waveguides. Cleaning and activation of surfaces, covalent binding of organosilanes, passivation of non-reacted surface groups, immobilization of recognition elements (i.e., aptamers), and subsequent passivation of reactive groups were all characterized with different techniques in order to obtain an optimized protocol to be transferred to the biosensor surface. The best performing functionalization protocol was finally implemented on a photonic chip (MMR biosensor) in order to verify the suitability of this protocol for biomarker detection. Aptamers were used as the recognition element of proteins related to inflammation (C-reactive protein or CRP [26]), viral infection (spike protein of the SARS-CoV-2 virus [27]), and thrombotic diseases (thrombin [28]).

## 2. Materials and Methods

### 2.1. Materials

Substrates (1 cm^2^) of c-Si (100) silicon (Si), thermally-grown silicon oxide (TG-SO) and silicon nitride on silicon (SiN) deposited by low-pressure chemical vapor deposition and photonic chips were produced in-house at the FBK-Microfabrication facility; 2-[Methoxy-(polyethyleneoxy)propyl]trimethoxysilane, 6–9 (PEG-s) was acquired from Fluorochem Ltd., Hadfield, UK; 3-Glycidyloxypropyl-trimethoxysilane (GPTMS), 3-mercaptopropyltrimethoxysilane (MPTMS), toluene anhydrous (99.8%), toluene, dithiothreitol (DTT), ethanolamine (EA), 6-mercapto-1-hexanol (MCH), C-reactive protein (CRP), thrombin from human plasma (Thr), bovine serum albumin (BSA), Tween 20, and all powders for buffer solutions were purchased from Merck Life Science S.r.l. (Milan, Italy). Ellman’s reagent, anti-CRP polyclonal goat IgG antibody horseradish peroxidase (HRP) conjugated and SuperSignal™ELISA Femto Substrate were from ThermoScientific (Waltham, MA, USA). All aptamers were produced by IDT Integrated DNA Technologies (Leuven, Belgium).

### 2.2. Setup of the Functionalization Protocol

**Figure 1:**
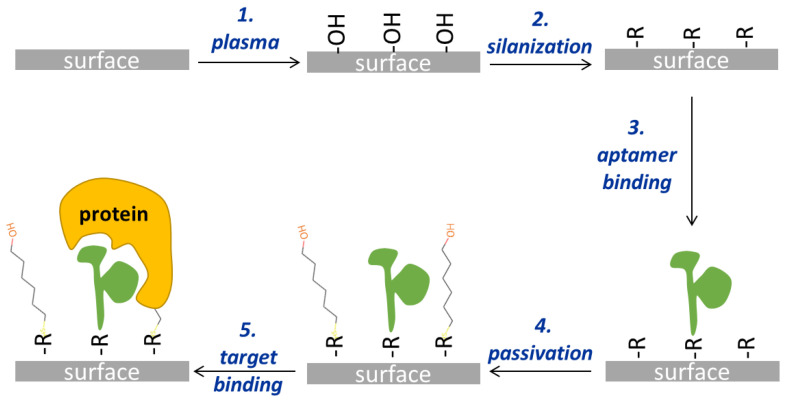
Schematic drawing of the four steps of the surface functionalization protocol leading to the target binding (fifth step). “R” refers to the silane residue, i.e., the epoxy group when GPTMS is used, and the thiol group when MPTMS is used.

The surface functionalization protocol comprises several steps, as illustrated in Figure 1. The first step involves a plasma treatment aimed both at cleaning the surface from carbon contamination and at activating the surface for the following step, i.e., silanization. Different plasma treatments (Table 1) were tested, employing either argon or oxygen gas. The argon plasma treatment was performed with the PDC-32G plasma cleaner (Harrick Scientific Corporation, New York, NY, USA), while for the oxygen plasma, the COL-1B-MF Colibrì (Gambetti, Milan, Italy) plasma cleaner was used.

In addition to plasma, different silanization protocols were also tested (Table 2; Step 2 in Figure 1). Solutions containing either GPTMS or MPTMS or a mixture of MPTMS and PEG-s were dissolved in toluene anhydrous and used to incubate the surfaces at 60 °C for 10 min. After three washes with toluene, the surfaces were dried under a stream of nitrogen and used for aptamer binding (Step 3). Alternatively, after washing with toluene and before drying with nitrogen, a sonication step of 10 min in ethanol absolute was performed. In Step 3, different concentrations of aptamers (listed in Table 3) were dissolved in a suitable buffer and made to react with the silanized surfaces for various times at room temperature.

Aminated aptamers were diluted in 50 mM sodium phosphate with 300 mM ionic strength and subjected to a thermal shock (1 min at 95 °C followed by several minutes in ice) just before use. A total of 30 µL of aptamer was added to the GPTMS-silanized surfaces positioned in a hybridization chamber for 3 h at room temperature. After this time, the surfaces were vigorously washed three times in the same buffer and passivated with 1 mM EA for 1 h, when required (Step 4, Figure 1).

Thiolated aptamers were reduced with DTT just before binding. Briefly, 10 mM DTT in 0.5 M sodium carbonate with a pH of 9 (coupling buffer or CB) was added to the aptamer solution for 1 h at room temperature and 400 rpm agitation. DTT was then removed with Amicon® Ultra-0.5 Centrifugal Filter Devices (Merck Life Science S.r.l.; Milan, Italy), and the final concentration of the aptamer was quantified by spectrophotometry (Nanodrop ND-1000 spectrophotometer, Nanodrop Technologies, Wilmington, DE, USA). Suitable concentrations of reduced aptamer (either fluorescently labeled or not) were used for surface binding. Then, 30 µL of reduced aptamer in CB was placed on the surfaces for 3 h at room temperature in a hybridization chamber to avoid evaporation. Surfaces were then washed, first in CB added with 0.1% Tween 20, next in CB, and finally in water, before moving to Step 4, i.e., passivation. Surfaces were immersed in a solution of 1 mM MCH in CB for different lengths of time at room temperature, and then two washes with CB and one with ultrapure water were performed. Finally, surfaces were dried in a stream of nitrogen and preserved at 4 °C until used. The final step of the protocol shown in Figure 1 (Step 5) consists of the recognition of the target protein operated by the aptamer on the surface.

### 2.3. Characterization of the Functionalization Steps

Every step of the functionalization protocol was characterized with several techniques, as described below.

The static contact angle (CA) was measured by depositing 2 µL of ultrapure water on at least four different areas of the surface. Images of the droplets were acquired with a CMOS camera and analyzed by Drop-Analysis, a plugin of ImageJ software [29]. Results are reported as averages and standard deviations.

Atomic force microscopy (AFM) was used to assess the morphology and roughness of both the plane surfaces (before and after the different treatments) and to characterize the structures fabricated on the photonic chips and their surfaces. AFM measurements were performed using a Cypher AFM (Oxford Instruments-Asylum Research, Santa Barbara, CA, USA) in AC mode in air, using OMCL-AC240TS Olympus cantilevers (Oxford Instruments GmbH Asylum Research, Wiesbaden, Germany), with a nominal force constant of 2 N/m and a nominal resonance frequency of 70 kHz. Acquired data were plane fitted, imaged, and analyzed with a custom ImageJ (version number 0.1.8) [29] distribution for AFM data analysis (AFMiJ: https://github.com/AFMiJ/AFMiJ, accessed on 26 March 2024).

The number of thiol groups on MPTMS silanized surfaces was quantified using Ellman’s assay. Ellman’s reagent (5,5′-dithiobis-(2-nitrobenzoic acid), DTNB) is a colorogenic chemical that reacts with sulfhydryl groups, producing a yellow-colored compound (2-nitro-5-thiobenzoic acid, TNB) with a maximum absorbance peak at 412 nm. Surfaces silanized with MPTMS were covered with a solution of Ellman’s reagent at 0.08 mg/mL concentration in reaction buffer (0.1 M sodium phosphate at pH 8, 1 mM EDTA) and left at room temperature for 15 min. The resulting solution was collected and measured with the Jasco V-550 spectrophotometer. Absorbance at 412 nm was used to calculate the number of sulfhydryl groups on the surfaces by comparison with a calibration curve, previously prepared by measuring known amounts of MPTMS solutions.

The X-ray photoelectron spectroscopy (XPS) analysis was performed on the differently treated surfaces with the Scienta ESCA 200 instrument equipped with a hemispherical analyzer and a monochromatic Al K*α* (1486.6 eV) X-ray source in transmission mode and with an angle between the normal to the sample surface and the analyzer axis of 0°. High-resolution spectra were acquired for each surface (Si 2p, O 1s, C 1s, and S 2s core lines). The XPS quantification was performed using the instrument sensitivity factors and the high-resolution spectra, while data were analyzed with the software described in Speranza and Canteri [30].

The binding of fluorescent aptamers was checked by fluorescence microscopy. The fluorescence of the plane surface was acquired with the Leica DMLA microscope (Leica Microsystems, Wetzlar, Germany), equipped with a mercury lamp and the fluorescence filter N2.1 (Leica Microsystems, Wetzlar, Germany). Surfaces were observed with a 20× objective and measured with a cooled CCD camera (DFC 420C, Leica Microsystems, Wetzlar, Germany). A matrix of 6 × 6 tiles, 537 µm wide and 404 µm high each, were acquired, controlling the microscope motorized stage through a BeanShell script running in MicroManager (version 1.4.23, micro-manager.org). The reconstruction of images as well as all the analyses were performed with the Fiji software [31] and Fiji custom macros. Fluorescent aptamers bound to the photonic chip were imaged with a Leica SP5-II confocal microscope, using a 63× objective in water. The acquired images were elaborated with the Fiji software (version 2.1.0, created by Johannes Schindelin, Albert Cardona and Pavel Tomancak) [31].

The chemiluminescence test was performed to assess the protein binding to its aptamer and to check the stability of the aptamer layer during the time period. Surfaces exposing a-CRP aptamers were submerged in a solution of 100 nM CRP in binding buffer (20 mM Hepes, 120 mM NaCl, 5 mM KCl, 1 mM CaCl_2_, 1 mM MgCl_2_, pH 7.3) and left to incubate for 30 min at room temperature. The excess of protein was removed by three washes with binding buffer before blocking the surfaces with a solution of BSA 1% *w*/*v* and Tween 20 0.1% *v*/*v* in binding buffer for 30 min at room temperature. The passivated surfaces were then incubated with an anti-CRP antibody HRP conjugated for 1 h at room temperature. Two washes with the solution of BSA 1% *w*/*v* and Tween 20 0.1% *v*/*v* in binding buffer and one final wash with binding buffer were then performed. Finally, the chemiluminescence signal was developed with the kit SuperSignal™ELISA Femto Substrate, measured using a ChemiDoc MP Imaging System (BioRad, Hercules, CA, USA). The measured signal was quantified using the Fiji software [31].

### 2.4. Optical Setup and Measurements 

**Figure 2:**
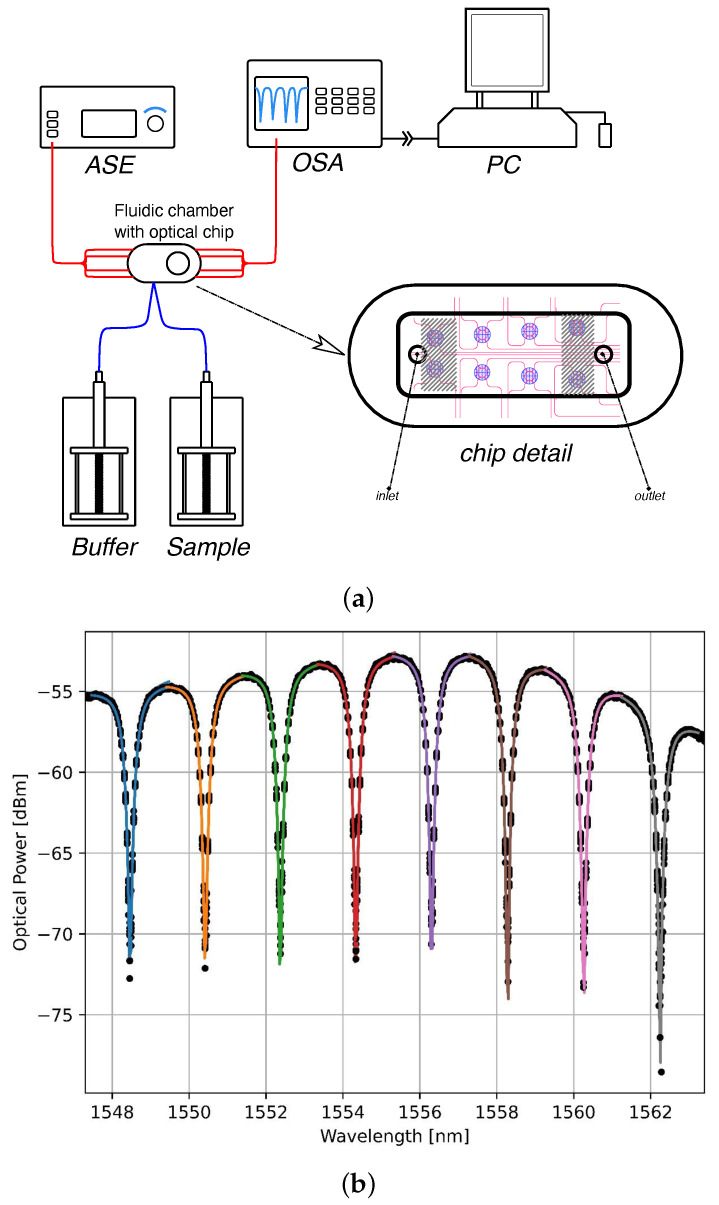
Optical measurement setup. (**a**) Schematic representation of the optical measurement setup (fluidic in blue, optical fibers in red) and magnification showing the fluidic chamber superimposed on a sketch of MRRs and optical waveguides (chip detail). The four central microrings (not hatched in the figure) were used for the binding measurements reported in this work. (**b**) MRR resonance spectrum showing the optical power (“through” direct output) as a function of the input wavelengths. The different colors refer to the different resonances.

The core of the optical setup (Figure 2) is the photonic chip, which comprises an array of two rows with four microring resonators (MRRs) each. The optical circuit used consists of four MRRs in an add–drop configuration. The optical waveguide and ring were designed for use in liquid solutions and waveguiding around 1.5 µm. The nominal width of the waveguides is 1100 nm. The fabrication process starts with the deposition of 250 nm of Si_3_N_4_ LPCVD silicon nitride on c-Si silicon substrates covered with 5 µm thick thermal oxide as bottom cladding. The waveguide circuit is then realized by optical lithography with an i-line stepper, followed by reactive ion etching of the SiN film. The whole circuit is then covered by a thin silicon nitride layer (LPCVD), which is used as the etch step for the definition of the sensor windows. Next, 2 µm of boronphosphorsilicon glass (BPSG) was deposited, followed by a thermal reflow process to form the top cladding and to obtain a smooth upper cladding. In a second lithographic process, the sensor windows were defined and etched by a combination of dry (reactive ion etching) and wet chemical etching in buffered hydrofluoric acid. The thin silicon nitride layer functions as the etch stop for the BPSG removal in the sensor areas. The SiN refractive index was 1.96 at 1550 nm, while the MRRs had an almost circular shape, slightly compressed in the coupling direction, with a diameter of 200 µm.

For the measurement protocol, already functionalized photonic chips were assembled into the fluidic chamber and connected to two motorized syringes delivering sample and buffer, respectively (Figure 2a). The fluidic chamber is superimposed on the array of eight MRRs of the photonic chip in order to maximize the exposure of the four central MRRs to the fluid flux, as shown in Figure 2a (chip detail). Once assembled, the biosensor was aligned to the optical input (amplified spontaneous emission light source or ASE in Figure 2a) and output (optical spectrum analyzer or OSA in Figure 2a). The light emitted by the ASE was routed by the input optical switch (1 × 4) alternatively into four fibers of the fiber array, which were directly coupled to the input waveguides of the four different MRRs used for the sensing. Four more fibers were coupled to the “through” (direct output) of the rings, so that the output optical switch (4 × 1) would sequentially send an output signal to the OSA. The OSA is a spectrum analyzer that provides power as a function of wavelength. The spectral range was analyzed between 1547 and 1563 nm, covering eight resonances (the rings have a free spectral range of 1.9 nm). To monitor the system dynamics, at each time step (occurring once per second), an MRR spectrum was acquired. Subsequently, the peaks of the resonances were fitted with Lorentzian curves (see Figure 2b), resulting in a dataset of peak positions (measured in picometers) as a function of time for the eight resonances. Finally, the datasets resulting from the fitting of the resonances were averaged to obtain a single representation of the resonance wavelength shift (expressed in picometers) over time. Real-time monitoring of the system dynamics was facilitated by a graphical representation displayed on a monitor (PC in Figure 2a).

Fluids were injected at 20 µL/min through the fluidic chamber; the running buffer (50 mM Tris-HCl, 150 mM KCl, 1 mM MgCl_2_ pH 7.4, with 0.05% Tween 20 added) was firstly flooded on the biosensor surface until reaching the stability of the signal, i.e., MRR resonance positions are stable in time; after that, a solution of 100 nM thrombin in the same buffer was injected for about 20 min before again fluxing the buffer.

## 3. Results and Discussion

The functionalization strategy selected for the MRR surface is based on the covalent binding of aptamers through organosilanes exposing epoxy or mercapto groups. Organosilanes are often used to functionalize the MRR surface [20] due to the oxidized silicon surface of these sensors. However, the organosilanes generally used are the amino- and epoxy-silanes. In this work, a comparison between a classically used epoxysilane and a mercaptosilane is presented and deeply characterized for each step of the functionalization process in order to select the best-performing molecule. Moreover, aptamers show several advantages, including their production via chemical synthesis, which allow for the introduction of selective chemical groups at the end of the nucleotide chain in order to obtain an oriented immobilization. This strategy is direct and quite simple, yielding reproducible and stable surface modifications. The selected functionalization of the sensor surface requires several steps (Figure 1), which were tuned and characterized.

### 3.1. Activation of Silicon Surfaces by Plasma Treatments

The first step of the functionalization protocol relies on the cleaning and activation of the silicon surface with the two plasma treatments illustrated in Table 1. To verify the treatment efficacy, the effect of both treatments on surfaces were characterized by CA, AFM, and XPS. CA measurement of the bare silicon nitride surface resulted in 47° ± 3°, while CA values were extremely low, i.e., below the instrumental resolution of 5°, for both the plasma conditions tested, indicating the formation of very hydrophilic surfaces with respect to the initial silicon nitride substrates, as expected. Plasma-treated surfaces were also analyzed by AFM, resulting in surfaces that were quite flat with a roughness similar to the original silicon surface (silicon surface roughness was 0.23 nm ± 0.01 nm), as shown in Figure S1 and Table 4. In addition to wettability, morphology, and roughness, chemical composition of the plasma-treated surfaces was measured by XPS (Table 4). Both p1 and p2 protocols gave good performances in terms of surface cleaning; however, the carbon removal was much better when the surfaces were treated with the argon plasma p1 with respect to the oxygen plasma p2, and therefore the p1 treatment was selected as the most efficient for the cleaning of the final optical devices. Moreover, as reported in Guarisco et al. [23], plasma treatments increase the percentage of oxygen with respect to bare silicon nitride surfaces, possibly because of an increased presence of exposed –OH groups (see Step 1 in Figure 1). Plasma treatments are therefore effective not only in cleaning but also in activating the silicon surfaces for the next functionalization steps.

### 3.2. Development of the Silanization Protocol

The second step for the setup of the functionalization protocol is based on the selection of the optimal silanization conditions and of the optimal silane terminal group for a suitable aptamer binding efficiency. Two different organosilanes were selected: (i) a silane with an epoxy group as residue able to covalently bind amino groups (GPTMS) and (ii) a silane with a thiol group as terminal residue, used to immobilize aptamers through disulfide bonds (MPTMS).

Concerning GPTMS, two concentrations (1% and 0.1% *v*/*v*) were selected for modifying silicon surfaces in wet conditions after the different plasma treatments (see Section 2.2). The efficacy of silanization was evaluated first in terms of wettability (Table 5), revealing quite a large increase in comparison with plasma-treated surfaces, but no significant differences among different plasma treatments, concentrations of silane solutions, or the final sonication step. Values of the contact angles were similar to those reported in the literature [32,33], ranging around 60°. For MPTMS silanization, similar conditions as for GPTMS were tested, i.e., two concentrations (1% and 0.1% *v*/*v*), different plasma treatments, and the final sonication step. Also, for MPTMS surfaces, the effect of the various treatments was checked for possible changes in wettability (Table 5). Contact angles of surfaces treated with p1 or p2 plasma and prepared with 1% MPTMS were similar, i.e., around 60°, indicating that the different plasma treatments do not influence the silanization step much. As for GPTMS, contact angle values measured for MPTMS are in good agreement with the literature [34,35,36]. On the contrary, p1 and p2 surfaces silanized with both 1% and 0.1% MPTMS but treated with a final sonication step presented reduced contact angle values, decreasing to below 50°, which correspond to a higher surface hydrophilicity.

**Figure 3:**
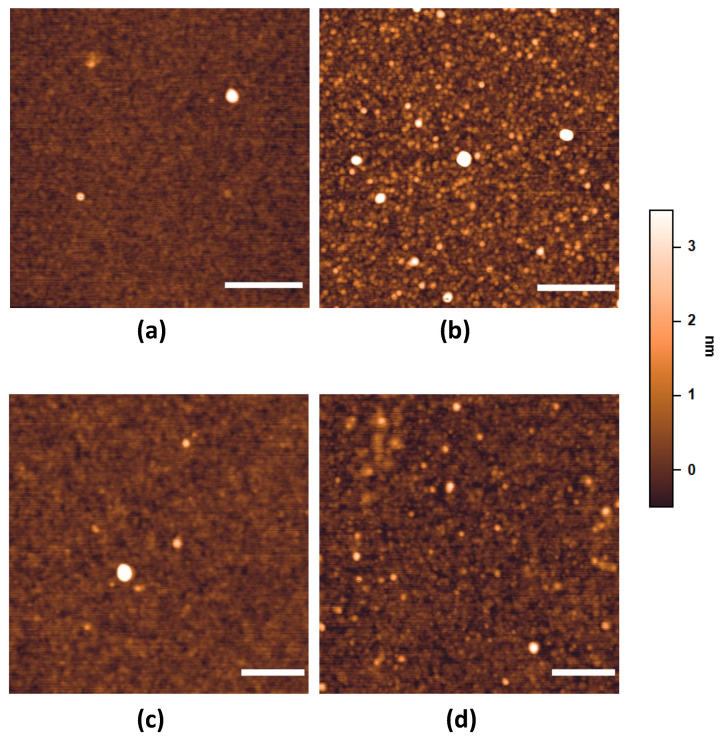
AFM images of plane silicon surfaces. (**a**) Silicon without treatment; (**b**) p1g01 treatment; (**c**) p1m01 treatment; (**d**) p1m1 treatment. The false color scale ranges from −0.5 to +3.5 nm, while scale bars represent 200 nm in (**a**,**b**), and 100 nm in (**c**,**d**).

The silanized surfaces were further characterized by AFM analysis, exploring changes in morphology and roughness due to the various conditions. Non-treated silicon was quite smooth (0.23 nm ± 0.01 nm), with some aggregates, possibly contamination particles, dispersed on the surface (2–3 particles/µm^2^, Figure 3a). In addition to aggregates, the morphology definitely changed after silanization, as clearly visible in Figure 3b, indicating that the reaction with GPTMS successfully occurred. The surface roughness also increased, passing from plasma-treated surfaces (Table 4) to surfaces silanized with 0.1% and with 1% GPTMS, while it decreased after sonication (Table 5). The surface p1g1 was the roughest, possibly because of silane molecules non-covalently bound to the surface but attached in the form of small aggregates. This hypothesis is confirmed by the sonication step, which was able to reduce the surface roughness to values similar to those of the p1g01 surface.

MPTMS surfaces were also analyzed in terms of morphology and roughness by AFM (Table 5 and Figure 3). The morphology of all surfaces was quite homogeneous, with few nanostructures distributed on the whole surface (Figure 3c,d). The surface modifications were evident by comparing the non-treated surface with the p1m01 surface and, even more, with the p1m1 surface (see Figure 3a,c,d), confirming that the silanization process occurred successfully. The sonication step instead influenced GPTMS more than it did MPTMS silanization (Table 5).

In addition to wettability and morphology, the amount of thiol groups on MPTMS surfaces were quantified with Ellman’s assay (Section 2.3). This test relies on Ellman’s reagent, a chemical that reacts with sulfhydryl groups to release a colored compound. This assay was developed for use in solution, but it was also adapted for the determination of thiols on surfaces [35,37]. The number of thiols was higher for surfaces modified with the higher amount of MPTMS, i.e., 1%, independently from the plasma treatment previously applied to the surface (Table 5). Moreover, the sonication step did not reduce this number much, which remains on the order of 10^14^/cm^2^. This amount is in good agreement with those reported for other organosilane films on silicon surfaces [38,39] and higher than the optimal number of aptamer molecules immobilized on the surface for the best target recognition performances [40].

The characterization of the different functionalization treatments for defining the most efficient in terms of coating, homogeneity, and surface reactivity was completed with the chemical analysis performed by XPS. The success of the functionalization with GPTMS is attested by the increase in carbon and oxygen percentages, i.e., the atoms present in the epoxy group (Table 5). The increase in the carbon percentage was evident in all the tested conditions, independently from the previous plasma treatment, while the percentage of oxygen was higher for surfaces treated with the oxygen plasma before silanization, as expected. Moreover, the core lines of C 1s showed the presence of a contribution compatible with epoxy groups, confirming that GPTMS efficiently functionalized the surfaces. Analogously, the analysis of the chemical composition of surfaces treated with MPTMS demonstrated that this silane coated the substrates in all the tested conditions (Table 5). In this case, the presence of sulfur due to the MPTMS molecule was evident for all substrates, with the sulfur relative percentage higher when surfaces were treated with a higher concentration of MPTMS, i.e., 1%.

Taken together, the data regarding the characterization of silanized surfaces suggest that the most promising silanization with GPTMS is p1g01, since this condition produces a homogeneous coating whose wettability, roughness, and chemical composition is compatible with a thin layer of molecules covering the entire surface. Concerning MPTMS, the most promising silanization is p1m1, as it presents more functional sulfydryl groups (see Table 5) together with a suitable morphology (Figure 3d), roughness (Table 5), and the presence of sulfur (Table 5). These two silanizations were therefore further tested for setting up the aptamer binding.

### 3.3. Binding of Aptamers on the Silanized Surfaces

**Figure 4:**
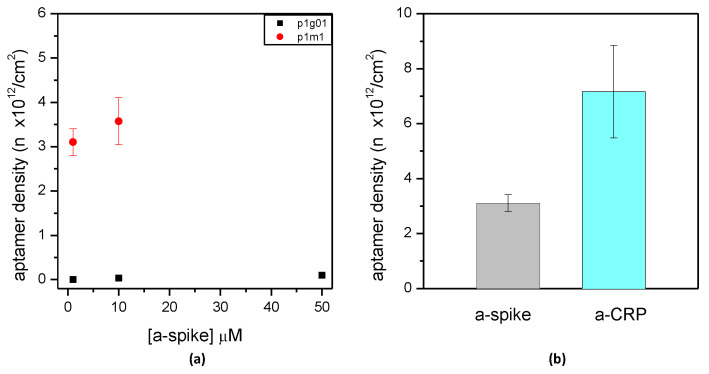
Density of aptamer molecules on silicon nitride plane surfaces at different concentrations of aptamers. (**a**) Amino-terminated or thiol-terminated and fluorescent a-spike aptamers bound at different concentrations to p1g01 (black squares) and p1m1 (red circles) surfaces, respectively. (**b**) Comparison between thiol-terminated and fluorescent a-spike and a-CRP aptamers bound to p1m1 at a 1 µM concentration. Means and standard deviations of at least three independent surfaces are reported.

The binding of aptamers was set up on the differently functionalized surfaces with fluorescent aptamers. The amount of aptamer molecules on the surfaces was calculated by comparing the fluorescence signals with known amounts of molecules, obtaining the aptamer density for the different protocols. First, the concentration of aptamers used for the immobilization step was varied (Figure 4). Both GPTMS- and MPTMS-based surfaces were tested for the binding of the a-spike aptamer, finding that the p1m1 silanization promoted the highest aptamer density (Figure 4a). Moreover, an increase of 10 times in the aptamer concentration, i.e., from 1 to 10 µM, led to a small increase in the aptamer density (Figure 4a). Therefore, it seems that a 1 µM a-spike aptamer concentration is a promising amount for a good binding on surfaces, as also confirmed by testing the a-CRP aptamer (Figure 4b). Based on these results, the functionalization protocol p1m1 was selected as the most promising for further optimization.

**Figure 5:**
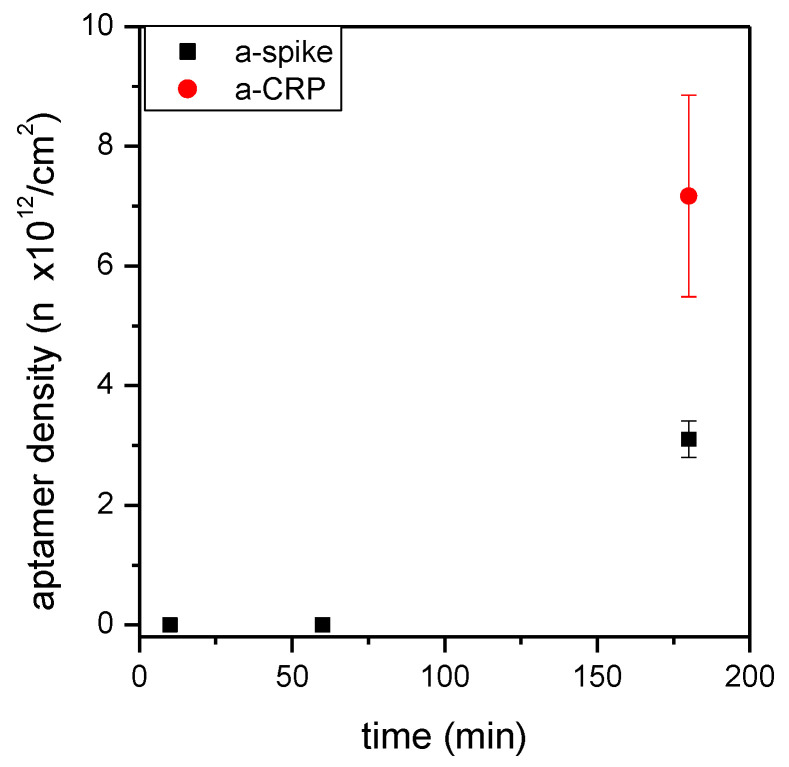
Setup of the aptamer binding on MPTMS silanized surfaces: time of reaction. Aptamer density on silicon nitride surfaces, which were functionalized as p1m1 and treated with 1 µM of a-spike or a-CRP aptamer at room temperature for different times.

In addition to the concentration, the length of time of the aptamer immobilization was varied (Figure 5). Using an aptamer solution of 1 µM concentration, three time periods were tested, i.e., 10 min, 1 h, and 3 h. Three hours of incubation was the time that provided the maximum density of aptamers on the surface.

It should be noted that the binding of aptamers through thiol chemistry requires a preliminary step for reducing the disulfide bonds possibly present, thus maximizing the presence of free –SH groups. Thiolated aptamers are indeed usually available in a form protected from spontaneous oxidation. The reduction step has to be performed just before use, lengthening the overall functionalization protocol. However, a certain binding activity towards surfaces exposing free –SH is also reported for molecules carrying non-reduced thiols [41]. To test this possibility, p1m1 surfaces were incubated with either reduced or non-reduced aptamers. The binding of the a-spike aptamer decreased around six times using non-reduced aptamers, i.e., from 3.1 ± 0.3 × 10^12^ (standard reduction step) to 0.5 ± 0.1 × 10^12^ molecules/cm^2^ (non-reduced aptamer), and the binding of the a-CRP aptamer was decreased by around two times, from 7.2 ± 1.7 × 10^12^ to 3.4 ± 0.1 × 10^12^ molecules/cm^2^. The reduction in aptamers therefore gave the best results in terms of surface density and was selected as the preferred condition for aptamer binding.

As mentioned above, the aptamer density on the surface was on the order of 10^12^ molecules/cm^2^ for both tested aptamers, in good agreement with the literature. It is reported that this surface density is optimal for the interaction of DNA aptamers with their target proteins [40]. The functionalization p1m1, followed by an incubation of 1 µM of reduced aptamer for 3 h at room temperature, were therefore selected as suitable conditions for all further experiments.

### 3.4. Passivation of the Functional Surfaces

**Figure 6:**
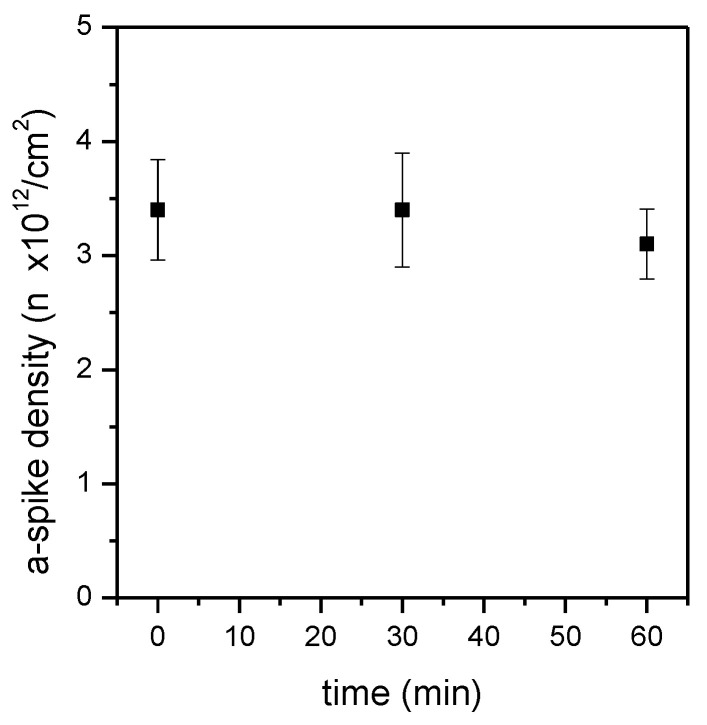
Surface density of the a-spike aptamer on p1m1 silicon nitride surfaces, passivated with 1 mM MCH for different lengths of time. Time 0 refers to non-passivated surfaces.

When aptamers are immobilized on the surface, not all available reactive groups are occupied by the DNA molecules, and the remaining free reactive groups can possibly interact with other molecules, such as with the aptamer target, during the following analysis. A passivation step is therefore essential to minimize the non-specific binding. Different treatments were evaluated, starting from the widely used passivating agent mercaptohexanol (MCH) [42]. The treatment with MCH was performed at various times (Figure 6) after binding of the a-spike aptamer. The aptamer density was not influenced by MCH passivation, remaining quite stable even after 30 or 60 min of incubation with MCH.

In addition to MCH, a strategy based on the use of PEG silane molecules was investigated. PEG-s was directly added to the silanization solution with the aim of distancing the thiol groups on the surface, filling the space among them with PEG-s, and decreasing the absolute number of thiol groups, while attempting to maintain a sufficient number of reactive groups for the successful aptamer binding. A further passivation step with MCH for 1 h was also tested. Unfortunately, the bound aptamer density decreased by more than a factor of 10 in this condition (Figure S2). The passivation with MCH was therefore preferred because it was compatible with the preservation of the optimal aptamer density.

In addition to passivation, the time stability of both the silane layer and the aptamer layer were investigated. Surfaces functionalized with the p1m1 treatment were stored at 4 °C for several days and tested for aptamer binding at fixed times (Figure S3a). A quite constant aptamer density was observed during time, evidencing that the silane layer is relatively stable at least for one week at 4 °C. The stability of the aptamer layer was instead verified as ability to bind aptamer targets. Surfaces with p1m1 functionalization and a-CRP aptamers were incubated at fixed times with a solution of CRP, which was then recognized by a specific a-CRP antibody conjugated with HRP. After adding a developing solution, a chemiluminescence signal was generated and converted into density of antibody on the surface by comparison with known amounts of the same antibody (Figure S3b). Different preservation conditions were tested, revealing that the storage of surfaces at 4 °C was the most suitable for the binding of protein target even after a couple of months. Therefore, surfaces functionalized with aptamers are quite stable up to two months if stored at 4 °C.

### 3.5. Functionalization Protocol Applied to Photonic Chips

**Figure 7:**
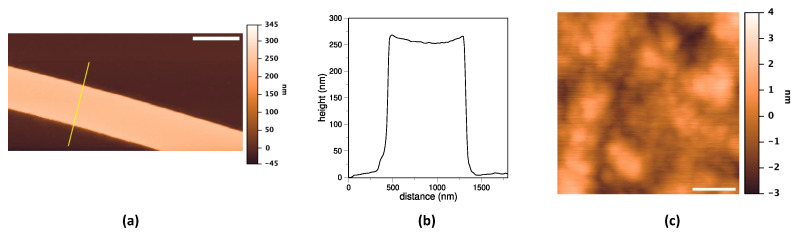
AFM analysis of photonic chip waveguides. (**a**) Typical image of a chip waveguide. Scale bar: 1000 nm. False color scale range from −45 to +345 nm. The yellow line indicates the section through which the height profile shown in panel (**b**) is drawn. (**c**) Representative image of the roughness of the top of the guide. Scale bar: 30 nm. False color scale from −3 to +4 nm.

The functionalization protocol p1m1 set up on plane surfaces was then applied to the surface of photonic chips. First of all, the morphology of the fabricated structures on the photonic chip, in particular of the waveguides, was verified by AFM (Figure 7). The resulting microring waveguide was about 1 µm wide and 250 ± 5 nm high, in agreement with the manufacturing process (see Section 2.4), while the surface roughness of the waveguide top was 1.0 ± 0.2 nm, and the bottom of the sensing window was 0.8 ± 0.4 nm, i.e., higher than the plane surface, i.e., 0.23 ± 0.01 nm, but still quite smooth.

**Figure 8:**
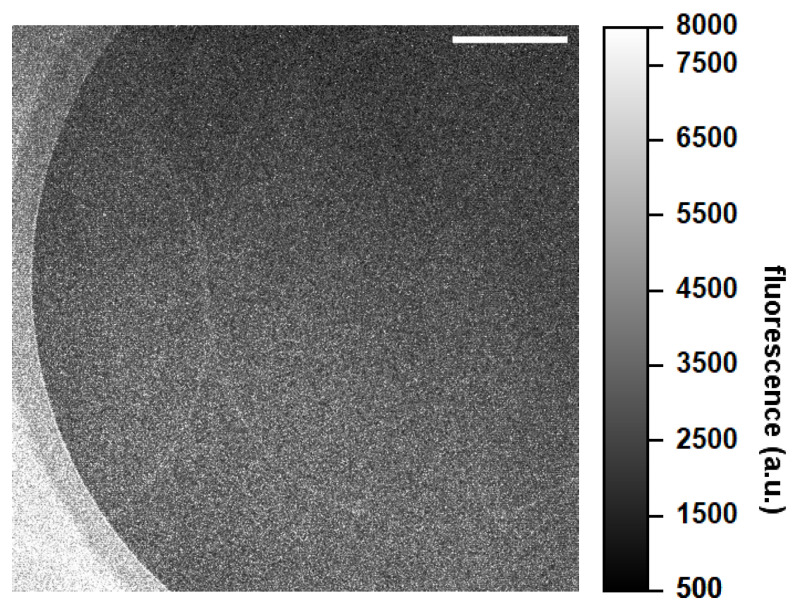
Confocal representative image of the fluorescent a-CRP aptamer immobilized on an optical microdevice. The area of the sensing window is shown, comprising waveguides that are more fluorescent than the surrounding area due to the difference in materials. Images are acquired with the 63× objective in liquid. The white bar refers to 50 µm.

The functionalization protocol, in particular the binding of aptamers, was also verified by immobilizing fluorescent aptamers and checking their presence by confocal microscopy (Figure 8). The device surface was homogeneously coated with the aptamers, resulting in a higher fluorescence signal in areas surrounding the MRR corresponding to the silicon oxide original substrate, while the sensing window areas, corresponding to silicon nitride, showed a slightly lower signal but were still homogeneous. The difference in substrate materials comprising the photonic chip is therefore reflected in the functionalization efficiency of the device and allows for the identification of waveguides, which showed a higher fluorescence signal with respect to the surrounding areas (Figure 8), confirming the good aptamer binding.

Since the functionalization protocol was valid for the photonic chips as well, a measure of protein binding was performed, as described in the next section.

### 3.6. Measures of Protein Targets with the Optical Setup

**Figure 9:**
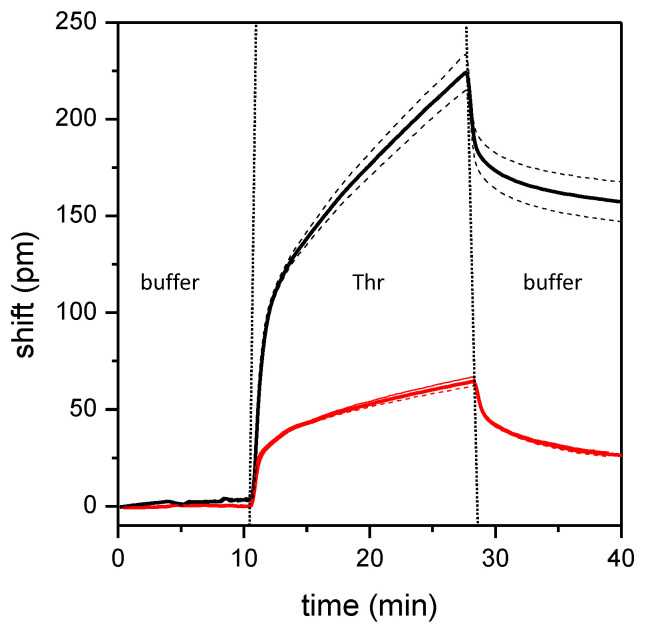
Optical detection of thrombin. Kinetics of thrombin binding on p1m1 functionalized photonic chip surfaces, with the a-Thr (black curve) or the NS (red curve) aptamer. Chip surfaces were passivated with 1 mM MCH. Both buffer and 100 nM thrombin solutions were added, with 0.05% Tween 20. Data are means of the four microrings, while standard deviations are represented as dashed curves.

The photonic chips were functionalized with the above optimized protocol (i.e., p1m1, Table 1 and Table 2) and then tested for their efficacy in real-time binding of protein targets. This proof of principle experiment was performed by selecting the well-known thrombin biomarker. Thrombin is a protein enzyme involved in the coagulation process, often analyzed as biomarker of thrombotic diseases. Several aptamers targeting Thr have been developed and characterized, including the a-Thr aptamer tested in this study [28,43,44]. In parallel with a-Thr, a different photonic chip was functionalized with the NS aptamer as a control for the possible non-specific binding on the functionalized surface. A flux of buffer was first injected on the functionalized chips to monitor a stable signal before adding 100 nM Thr spiked in the same buffer (Figure 9). Thr was found to quickly bind the specific aptamer, and an evident increase in the signal was observed (black curve in Figure 9). On the contrary, when Thr is fluxed on the chip functionalized with the non-specific aptamer, only a small signal increment was visible (red curve, Figure 9). This small signal could be ascribed to a non-perfect passivation of the chip surfaces, which still can adsorb some Thr molecules, even after passivation with MCH.

The last step of the optical measurement corresponds to a final buffer injection, which results in a decrease in the signal as a consequence of Thr unbinding for both tested aptamers. However, in the presence of the specific aptamer, about 71% of the adherent Thr is retained on the surface, whereas, when the non-specific aptamer is used, about 40% of Thr is still present after the unbinding step. These results suggest that the binding of thrombin to the a-Thr aptamer is specific and that only a small part of this binding could possibly be ascribed to Thr molecules adsorbed by the surface, while most of them are captured by the specific aptamer. Even if there is still room for improving the specificity of measurements, the simple functionalization strategy explored in this paper gave promising results in terms of surface stability and reproducibility. This preliminary experiment shows good potential of this MRR-based biosensor for biomarker detection in a point-of-care perspective.

## 4. Conclusions

The appealing possibility of performing liquid biopsy on-chip with the direct label-free detection of significant biomarkers was explored starting with biosensor surface functionalization. Among the several existing functionalization strategies, a protocol based on aptamer immobilization on a mercaptosilane layer was selected and tested. All steps of the surface functionalization protocol were characterized, finding that the optimal plasma treatment included argon gas, the best silanization reagent was MPTMS at 1% concentration, followed by incubation of 1 µM aptamer for 3 h, which was the most suitable protocol for aptamer immobilization, while a passivation step with 1 mM MCH was sufficient to discriminate the specific binding of a target protein. The aptamer functionalization was homogeneous, reproducible, and allowed stable surfaces to capture a good amount of protein biomarker. The surface functionalization protocol was first optimized on plane surfaces, then applied to the biosensor surfaces in order to verify the best conditions for the detection of biomarkers. Preliminary detection tests were performed with a photonic chip based on microring resonators for the detection of the well-known protein biomarker thrombin. The comparison between the specific a-Thr aptamer functionalization and a nonsense control aptamer allowed us to clearly discriminate the correct target. These promising results can be considered as a starting point for the construction of POC devices for on-chip liquid biopsy.

## Figures and Tables

**Table 1:** Plasma treatments tested on the plane surfaces.

Plasma Treatment Name	Gas	Power (W)	Time (min)
p1	argon	10.5	1
p2	oxygen	50	2

**Table 2:** Silanization treatments: type and amount of silane used for the functionalization step and presence of a further sonication step.

Surface Name	Plasma Treatment	Silanization Name	Silane	% *v*/*v*	Sonication
p1g1	p1	g1	GPTMS	1	-
p1g1s	p1	g1s	GPTMS	1	10’
p1g01	p1	g01	GPTMS	0.1	-
p1g01s	p1	g01s	GPTMS	0.1	10’
p2g1	p2	g1	GPTMS	1	-
p2g1s	p2	g1s	GPTMS	1	10’
p2g01s	p2	g01s	GPTMS	0.1	10’
p1m1	p1	m1	MPTMS	1	-
p1m1s	p1	m1s	MPTMS	1	10’
p1m01	p1	m01	MPTMS	0.1	-
p1m01s	p1	m01s	MPTMS	0.1	10’
p2m1	p2	m1	MPTMS	1	-
p2m1s	p2	m1s	MPTMS	1	10’
p2m01	p2	m01	MPTMS	0.1	-
p2m01s	p2	m01s	MPTMS	0.1	10’

**Table 3:** List of aptamers tested. All aptamers were DNA-based, purchased with one of the modifications listed in column “5′ modification”. Some amounts of aptamers were also modified at the 3′ end with a fluorescent label (TAMRA). All modifications are referred to using the standard nomenclature of the manufacturer. CRP: C-reactive protein; Spike: SARS-CoV-2 spike protein; Thr: thrombin; NS: nonsense aptamer.

Name	Sequence (5′–3′)	Target	5′ Modification	3′ Modification
a-CRP	CGA AGG GGA TTC GAG GGG TGA TTG CGT GCT CCA TTT GGT G	CRP	5ThioMC6-D/	/36-TAMSp/
a-spike	CAG CAC CGA CCT TGT GCT TTG GGA GTG CTG GTC CAA GGG CGT TAA TGG ACA	Spike protein	/5AmMC12/; /5ThioMC6-D/	/36-TAMSp/
a-Thr	AG TCC GTG GTA GGG CAG GTT GGG GTG ACT	Thrombin	/5ThioMC6-D/	
NS	ATC TAC GAA TTC ATC AGG	-	/5ThioMC6-D/	

**Table 4:** Elemental composition (%) determined by XPS analysis and average roughness of silicon surfaces with different plasma treatments. For XPS analysis, the take-off angle was 0°, and the error does not exceed 1–2% of the reported value.

	Elemental Composition			
**Surfaces**	**O %**	**C %**	**Si %**	**Roughness (nm)**
p1	31.1	-	68.9	0.24 ± 0.04
p2	41.1	5.3	53.6	

**Table 5:** First column: surface acronyms as defined in Table 2. Second column: elemental composition (%) determined by XPS analysis of silicon surfaces with different functionalization treatments at a take-off angle of 0°. The error does not exceed 1–2% of the reported value. Third column: average roughness measured by AFM. Fourth column: wettability measured by contact angle, reported as means and standard deviations of water contact angle. Last column: number of thiol groups on MPTMS treated surfaces measured with Ellman’s assay.

Surface	Elemental Composition				Roughness	Contact Angle	Thiol Density
(GPTMS)	O %	C %	Si %	S (%)	(nm)	(°)	(n × 10^14^/cm^2^)
p1g1					3.6 ± 2.8	57.3 ± 1.1	-
p1g1s	30.6	22.4	47.0	-	0.7 ± 0.1	58.0 ± 1.2	-
p1g01	31.7	18.0	50.3	-	0.7 ± 0.1	62.6 ± 2.0	-
p1g01s	30.9	19.5	49.6	-	0.35 ± 0.03	52.5 ± 0.7	-
p2g1	47.5	15.3	37.2		1.1 ± 0.7	55.7 ± 1.3	-
p2g1s	37.0	20.1	42.9	-	0.50 ± 0.04	52.2 ± 0.7	-
p2g01s	36.3	21.5	42.2	-	0.43 ± 0.03	52.0 ± 1.1	-
**(MPTMS)**							
p1m1	29.3	15.7	52.4	2.6	0.70 ± 0.06	60.1 ± 2.3	4.0 ± 0.9
p1m1s	29.8	15.9	52.8	1.5	0.9 ± 0.1	46.0 ± 2.7	3.6 ± 0.2
p1m01							1.1
p1m01s	28.7	13.2	56.1	2.0	0.61 ± 0.06	44.6 ± 2.3	0.5
p2m1						60.2 ± 2.1	4.2 ± 0.6
p2m1s	35.6	15.5	45.6	3.3	0.82 ± 0.07	45.8 ± 2.5	2.5 ± 0.5
p2m01							1.0
p2m01s	36.0	14.0	48.5	1.5	0.7 ± 0.1	45.3 ± 1.0	0.2

## Data Availability

The data presented in this study are available from the corresponding author upon request.

## Supplementary Materials

The following are available online at https://www.mdpi.com/article/10.3390/s24103107/s1: Figure S1. Surface roughness of plasma-treated surfaces; Figure S2. Surface density of the a-spike aptamer on differently passivated surfaces; and Figure S3. Surface stability of silanization and aptamer layer.

## References

[1] Bao Y., Zhang D., Guo H., Ma W. (2024). Beyond blood: Advancing the frontiers of liquid biopsy in oncology and personalized medicine. Cancer Sci..

[2] Braig Z.V. (2022). Personalized medicine: From diagnostic to adaptive. Biomed. J..

[3] Ignatiadis M., Sledge G.W., Jeffrey S.S. (2021). Liquid biopsy enters the clinic—Implementation issues and future challenges. Nat. Rev. Clin. Oncol..

[4] Duque G., Manterola C., Otzen T., Arias C., Palacios D., Mora M., Galindo B., Holguín J.P., Albarracín L. (2022). Cancer Biomarkers in Liquid Biopsy for Early Detection of Breast Cancer: A Systematic Review. Clin. Med. Insights Oncol..

[5] Patel A., Patel S., Patel P., Tanavde V., Tanavde V. (2022). Saliva Based Liquid Biopsies in Head and Neck Cancer: How Far Are We from the Clinic?. Front. Oncol..

[6] Napione L. (2021). Integrated Nanomaterials and Nanotechnologies in Lateral Flow Tests for Personalized Medicine Applications. Nanomaterials.

[7] Potrich C., Palmara G., Frascella F., Pancheri L., Lunelli L. (2024). Innovative Detection of Biomarkers Based on Chemiluminescent Nanoparticles and a Lensless Optical Sensor. Biosensors.

[8] Pittman T.W., Decsi D.B., Punyadeera C., Henry C.S. (2023). Saliva-based microfluidic point-of-care diagnostic. Theranostics.

[9] Tadimety A., Closson A., Li C., Yi S., Shen T., Zhang J.X.J. (2018). Advances in liquid biopsy on-chip for cancer management: Technologies, biomarkers, and clinical analysis. Crit. Rev. Clin. Lab. Sci..

[10] Moro G., Fratte C.D., Normanno N., Polo F., Cinti S. (2023). Point-of-Care Testing for the Detection of MicroRNAs: Towards Liquid Biopsy on a Chip. Angew. Chem. Int. Ed..

[11] Liu J., Geng Z., Fan Z., Liu J., Chen H. (2019). Point-of-care testing based on smartphone: The current state-of-the-art (2017–2018). Biosens. Bioelectron..

[12] Haghayegh F., Salahandish R., Zare A., Khalghollah M., Sanati-Nezhad A. (2022). Immuno-biosensor on a chip: A self-powered microfluidic-based electrochemical biosensing platform for point-of-care quantification of proteins. Lab Chip.

[13] Lai Z.X., Wu C.C., Huang N.T. (2022). A Microfluidic Platform with an Embedded Miniaturized Electrochemical Sensor for On-Chip Plasma Extraction Followed by In Situ High-Sensitivity C-Reactive Protein (hs-CRP) Detection. Biosensors.

[14] Harpak N., Borberg E., Raz A., Patolsky F. (2022). The “Bloodless” Blood Test: Intradermal Prick Nanoelectronics for the Blood Extraction-Free Multiplex Detection of Protein Biomarkers. ACS Nano.

[15] Rho D., Breaux C., Kim S. (2020). Label-Free Optical Resonator-Based Biosensors. Sensors.

[16] Soler M., Huertas C.S., Lechuga L.M. (2019). Label-free plasmonic biosensors for point-of-care diagnostics: A review. Expert Rev. Mol. Diagn..

[17] Ciminelli C., Campanella C.M., Dell’Olio F., Campanella C.E., Armenise M.N. (2013). Label-free optical resonant sensors for biochemical applications. Prog. Quantum Electron..

[18] Vollmer F., Arnold S. (2008). Whispering-gallery-mode biosensing: Label-free detection down to single molecules. Nat. Methods.

[19] Chalyan T., Potrich C., Schreuder E., Falke F., Pasquardini L., Pederzolli C., Heideman R., Pavesi L. (2019). AFM1 Detection in Milk by Fab’ Functionalized Si_3_N_4_ Asymmetric Mach-Zehnder Interferometric Biosensors. Toxins.

[20] Steglich P., Rabus D.G., Sada C., Paul M., Weller M.G., Mai C., Mai A. (2022). Silicon Photonic Micro-Ring Resonators for Chemical and Biological Sensing: A Tutorial. IEEE Sens. J..

[21] Komikawa T., Tanaka M., Yanai K., Johnson B.R.G., Critchley K., Onodera T., Evans S.D., Toko K., Okochi M. (2020). A bioinspired peptide matrix for the detection of 2,4,6-trinitrotoluene (TNT). Biosens. Bioelectron..

[22] Loyez M., Adolphson M., Liao J., Yang L. (2023). From Whispering Gallery Mode Resonators to Biochemical Sensors. ACS Sens..

[23] Guarisco M., Gandolfi D., Guider R., Vanzetti L., Bartali R., Ghulinyan M., Cretich M., Chiari M., Bettotti P., Pavesi L. (2017). A new aptamer immobilization strategy for protein recognition. Sens. Actuators B.

[24] Pilvenyte G., Ratautaite V., Boguzaite R., Samukaite-Bubniene U., Plausinaitis D., Ramanaviciene A., Bechelany M., Ramanavicius A. (2023). Molecularly imprinted polymers for the recognition of biomarkers of certain neurodegenerative diseases. J. Pharm. Biomed. Anal..

[25] Arnfinnsdottir N.B., Chapman C.A., Bailey R.C., Aksnes A., Stokke B.T. (2020). Impact of Silanization Parameters and Antibody Immobilization Strategy on Binding Capacity of Photonic Ring Resonators. Sensors.

[26] Wu B., Jiang R., Wang Q., Huang J., Yang X., Wang K., Li W., Chen N., Li Q. (2016). Detection of C-reactive protein using nanoparticle-enhanced surface plasmon resonance using an aptamer-antibody sandwich assay. Chem. Commun..

[27] Song Y., Song J., Wei X., Huang M., Sun M., Zhu L., Lin B., Shen H., Zhu Z., Yang C. (2020). Discovery of Aptamers Targeting Receptor-Binding Domain of the SARS-CoV-2 Spike Glycoprotein. Anal. Chem..

[28] Tasset D.M., Kubik M.F., Steiner W. (1997). Oligonucleotide inhibitors of human thrombin that bind distinct epitopes. J. Mol. Biol..

[29] Schneider C.A., Rasband W.S., Eliceiri K.W. (2012). NIH Image to ImageJ: 25 years of image analysis. Nat. Methods.

[30] Speranza G., Canteri R. (2019). RxpsG a new open project for Photoelectron and Electron Spectroscopy data processing. SoftwareX.

[31] Schindelin J., Arganda-Carreras I., Frise E., Kaynig V., Longair M., Pietzsch T., Preibisch S., Rueden C., Saalfeld S., Schmid B. (2012). Fiji: An open-source platform for biological-image analysis. Nat. Methods.

[32] Elender G., Kühner M., Sackmann E. (1996). Functionalisation of Si/SiO_2_ and glass surfaces with ultrathin dextran films and deposition of lipid bilayers. Biosens. Bioelectron..

[33] Wong A.K.Y., Krull U.J. (2005). Surface characterization of 3-glycidoxypropyltrimethoxysilane films on silicon-based substrates. Anal. Bioanal. Chem..

[34] Senkevich J.J., Yang G.R., Lu T.M. (2002). Thermal stability of mercaptan terminated self-assembled multilayer films on SiO_2_ surfaces. Colloids Surf. A Physicochem. Eng. Asp..

[35] Chen W.H., Tseng Y.T., Hsieh S., Liu W.C., Hsieh C.W., Wu C.W., Huang C.H., Lin H.Y., Chen C.W., Lin P.Y. (2014). Silanization of solid surfaces via mercaptopropylsilatrane: A new approach of constructing gold colloid monolayers. RSC Adv..

[36] Artusio F., Fumagalli F., Bañuls-Ciscar J., Ceccone G., Pisano R. (2020). General and adaptive synthesis protocol for high-quality organosilane self-assembled monolayers as tunable surface chemistry platforms for biochemical applications. Biointerphases.

[37] Bass J.D., Katz A. (2006). Bifunctional Surface Imprinting of Silica: Thermolytic Synthesis and Characterization of Discrete Thiol-Amine Functional Group Pairs. Chem. Mater..

[38] Santini G.C., Potrich C., Lunelli L., Pasquardini L., Vaghi V., Pederzolli C. (2014). Innovative microRNA purification based on surface properties modulation. Colloids Surf. B Biointerfaces.

[39] Pasquardini L., Lunelli L., Potrich C., Marocchi L., Fiorilli S., Vozzi D., Vanzetti L., Gasparini P., Anderle M., Pederzolli C. (2011). Organo-silane coated substrates for DNA purification. Appl. Surf. Sci..

[40] Simon L., Bognár Z., Gyurcsányi R.E. (2020). Finding the Optimal Surface Density of Aptamer Monolayers by SPR Imaging Detection-based Aptamer Microarrays. Electroanalysis.

[41] Rogers Y.H., Jiang-Baucom P., Huang Z.J., Bogdanov V., Anderson S., Boyce-Jacino M.T. (1999). Immobilization of Oligonucleotides onto a Glass Support via Disulfide Bonds: A Method for Preparation of DNA Microarrays. Anal. Biochem..

[42] Pasquardini L., Pancheri L., Potrich C., Ferri A., Piemonte C., Lunelli L., Napione L., Comunanza V., Alvaro M., Vanzetti L. (2015). SPAD aptasensor for the detection of circulating protein biomarkers. Biosens. Bioelectron..

[43] Pasquardini L., Berneschi S., Barucci A., Cosi F., Dallapiccola R., Insinna M., Lunelli L., Conti G.N., Pederzolli C., Salvadori S. (2013). Whispering gallery mode aptasensors for detection of blood proteins. J. Biophotonics.

[44] Riccardi C., Napolitano E., Platella C., Musumeci D., Montesarchio D. (2021). G-quadruplex-based aptamers targeting human thrombin: Discovery, chemical modifications and antithrombotic effects. Pharmacol. Ther..

