# Supplementary material for: Optimization of Surface Functionalizations for Ring Resonator-Based Biosensors
Source: Sensors (Basel). 2024 May 14;24(10):3107. doi: 10.3390/s24103107 (PMC11124806; doi:10.3390/s24103107)
Supplement: Supplementary file 1 [file sensors-24-03107-s001.zip › sensors-2961256-supplementary.pdf]

Surface roughness of plasma-treated surfaces

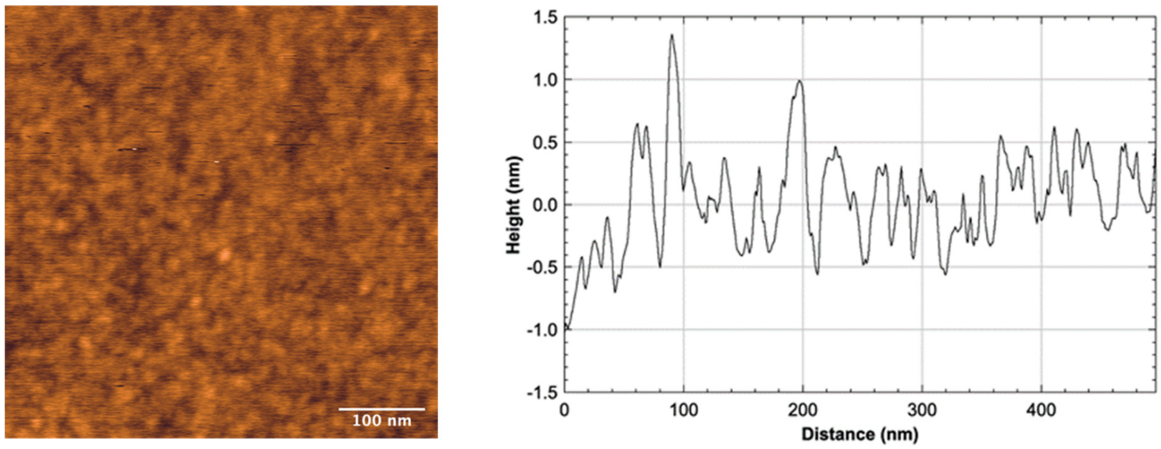

(a) (b)

Figure S1. AFM image (a) and a typical height profile (b) of silicon surface treated with p1 plasma. False color scale in (a) ranges from - 1.5 to + 4.0 nm, while the white bar refers to 100 nm.

Surface density of a-spike aptamer on differently passivated surfaces

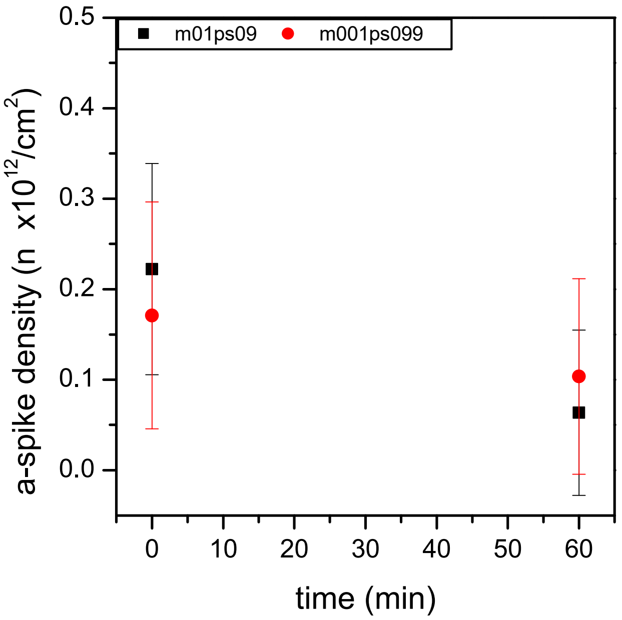

Figure S2. Surfaces silanized with a mixture of MPTMS and PEG-silane at 0.1/0.9% v/v (m01ps09, black squares) or 0.01/0.99% v/v (m001ps099, red circles). These surfaces were used for aptamer binding without further treatments (0 min) or after passivating for 1h with 1 mM MCH (60 min).

Stability of the functionalized surfaces

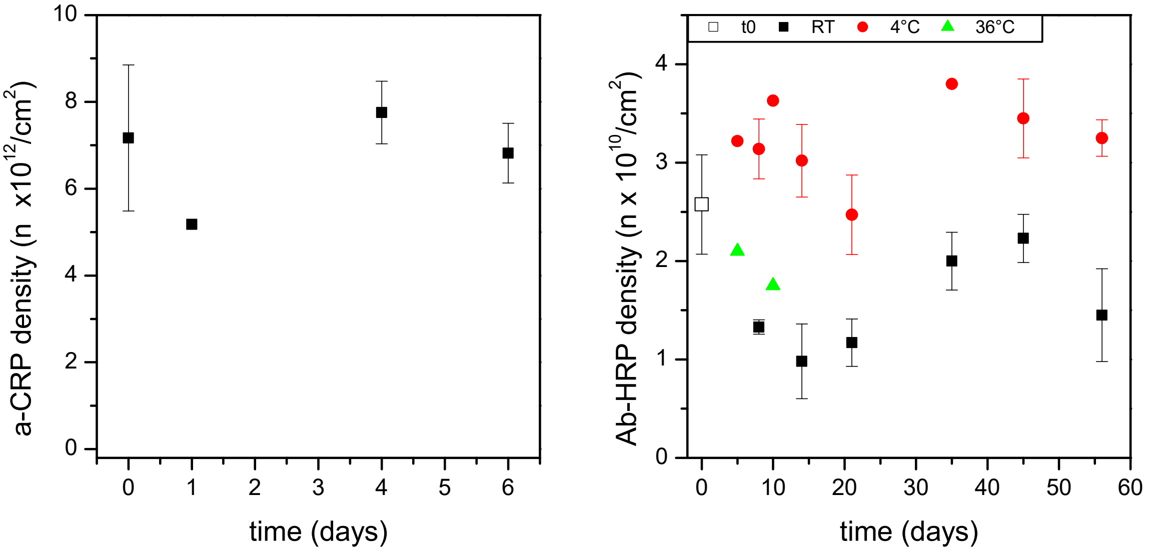

(a) **(b)**

Figure S3. Surface stability of silanization (a) and aptamer layer (b). Panel (a): p1m1 silanized surfaces were kept at +4°C for different time before being used for fluorescent a-CRP aptamer binding. Panel (b): p1m1 surfaces treated with a-CRP aptamer and passivated with MCH were kept at room temperature (black solid squares) or at +4°C (red circles) or at 36°C (green triangles) for several days before being used for the specific recognition of the aptamer target (CRP). The black open square represent surfaces measured in the same day of preparation (t = 0). CRP was detected in chemiluminescence by an a-CRP specific antibody HRP conjugated. Means and standard deviations are shown.
